# The Predictive Value of Clinical and Systemic Inflammatory Biomarkers in Emergency Colic Cancer Surgery: A Retrospective Study

**DOI:** 10.3390/jcm15041627

**Published:** 2026-02-20

**Authors:** Adrian Marius Silaghi, Crenguta Sorina Serboiu, Dragos Serban, Vlad Denis Constantin, Corneliu Tudor, Ion Motofei, Gebran Hussein, Paul Lorin Stoica, Marina Ionela Nedea, Ana Maria Dascalu, Tudor Mihai Badescu

**Affiliations:** 1 Doctoral School, “Carol Davila” University of Medicine and Pharmacy Bucharest, 020021 Bucharest, Romania; adrian-marius.silaghi@drd.umfcd.ro (A.M.S.); ana.dascalu@umfcd.ro (A.M.D.); 2 Department of General Surgery, St. Pantelimon Emergency Clinical Hospital, 021659 Bucharest, Romania; vlad.constantin@umfcd.ro (V.D.C.); ion.motofei@umfcd.ro (I.M.); 3 Faculty of Medicine, “Carol Davila” University of Medicine and Pharmacy Bucharest, 020021 Bucharest, Romania; sorina.serboiu@umfcd.ro; 4 4th Department of General Surgery, Emergency University Hospital Bucharest, 050098 Bucharest, Romania; 5 Department of General Surgery, Emergency County Hospital, 130095 Targoviste, Romania; 6 Faculty of Pharmacy, “Carol Davila” University of Medicine and Pharmacy Bucharest, 020021 Bucharest, Romania; marina.nedea@umfcd.ro; 7 Faculty of Medicine, “Lucian Blaga” University of Sibiu, 550169 Sibiu, Romania; tudor.badescu@ulbsibiu.ro

**Keywords:** colon cancer, emergency surgery, systemic inflammation biomarkers, C-reactive protein (CRP), the neutrophil-to-lymphocyte ratio (NLR), platelet-to-lymphocyte ratio (PLR), complications, anastomotic leak (AL)

## Abstract

**Background/Objectives**: Emergency surgery for complicated colon cancer carries high morbidity and mortality, largely driven by systemic inflammation and organ dysfunction. This study aims to investigate the predictive value of preoperative inflammatory biomarkers for postoperative outcomes. **Methods:** We retrospectively analyzed 219 patients undergoing emergency surgery for complicated colon cancer. Patients were classified as uncomplicated (*n* = 164) or complicated (Clavien–Dindo ≥ IIIA; *n* = 55). Preoperative clinical data, comorbidity indices, laboratory values, and inflammatory markers: C-reactive protein (CRP), neutrophil-to-lymphocyte ratio (NLR), platelet-to-lymphocyte ratio (PLR), and systemic immune-inflammation index (SII) were assessed. Logistic regression and ROC (Receiver Operating Characteristic) curves analyses identified predictors of Clavien Dindo complications graded as IIIA or higher, anastomotic leak (AL), and in-hospital mortality. **Results:** Most patients included in the study were males (75.02%), with a mean age of 69.63 (±11.54) years. Patients included in the complicated group had higher comorbidity burden, ASA (American Society of Anesthesiologists) grade, rates of diabetes, organ failure, and systemic inflammatory response. All inflammatory biomarkers were significantly elevated in the complicated group (*p* < 0.001). CRP (>62.8 mg/dL), NLR (>6.89), and PLR (>334.2) showed good discrimination for Clavien Dindo complications graded as IIIA or higher, with AUC (area under curve) ranging from 0.726 to 0.799. A multivariable model including Charlson Comorbidity Index (CCI), CRP, PLR, and diabetes predicted Clavien–Dindo ≥ IIIA complications with excellent accuracy (AUC 0.870). PLR, creatinine, and diabetes independently predicted AL (AUC 0.834). Mortality (20.5%) was strongly associated with peritonitis, CRP, and NLR (AUC 0.891). **Conclusions:** Preoperative inflammatory biomarkers, combined with comorbidity and renal function, reliably predict adverse outcomes after emergency colon cancer surgery. Multivariate models may be useful for early risk stratification and support individualized perioperative management.

## 1. Introduction

Colon cancer represents one of the most frequent causes of cancer-related mortality worldwide, accounting for approximately one million deaths in 2020 [1]. Surgical resection remains the main therapeutic approach in association with adjuvant systemic chemotherapy [2]. Implementation of a screening program based on regular colonoscopy, with early detection [3] and managed in a multidisciplinary team, has led to an increase in 5-year survival rates to 60–65% [4]. However, between 10% and 30% of patients present in emergency settings with complications such as bowel obstruction, hemorrhage or local perforation [5]. These acute manifestations are associated with increased postoperative morbidity, poor prognosis, reduced quality of life, and significantly higher medical costs [6,7].

In the emergency setting, surgical management of colon cancer often includes Hartmann’s procedure, typically performed on the left side of colon [8]. Other approaches may involve a diverting ostomy (if severe abdominal sepsis is present or if the resection is not feasible) [9] or segmental resection associated with primary anastomosis in selected patients [10]. The presence of organ dysfunction, severe comorbidities, hemodynamic instability or anemia increase postoperative morbidity, with complication rates reaching up to 42%, compared to less than 20% in elective surgery [11].

Common postoperative complications include anastomotic leakage (AL), prolonged ileus, surgical site infections, and stomal ischemia [12]. These events are associated with delayed recovery and can postpone the initiation of adjuvant therapy [13]. Surgical site infections (SSIs) are the most common postoperative complication after colorectal surgery, varying between 15 and 30% in different studies, particularly higher in emergency presentations, causing pain and suffering to patients. In addition, this complication has been associated with negative economic impact, increased morbidity, extended postoperative hospital stay, readmission, sepsis, and death [14,15]. Multiple predictive models were correlated with adverse postoperative outcomes, from physiological scoring systems and molecular markers to machine learning algorithms. While inflammatory markers (e.g., NLR, PLR, SII) specifically measure the immune response, other methods incorporate nutritional status, patient comorbidities, or tumor biology to provide a more comprehensive risk assessment, but more difficult to be used on emergency presentations [16,17]. Combined scores, such as the Glasgow Prognostic Score (GPS) or Prognostic Nutritional Index (PNI), integrate inflammation markers with serum albumin levels to better predict complications. The ACS NSQIP (American College of Surgeons National Surgery Quality Improvement Program) risk calculator is used to estimate patient-specific risks of postoperative complications [18].

In the context of emergency surgery and postoperative complications, a massive release of proinflammatory cytokines has been documented [19]. In addition, peritoneal contamination with bacteria and malignant cells creates a pro-oncogenic environment, leading to higher local recurrence rates and low 5-year survival [20].

Assessment of serological biomarkers reflecting the systemic inflammatory syndrome, such as the neutrophil-to-lymphocyte ratio (NLR), neutrophil-to-monocyte ratio (NMR), platelet-to-lymphocyte ratio (PLR) and the Systemic Immune-Inflammation Index (SII), had been used to assess the risk of recurrence and overall survival in several gastrointestinal malignancies, including gastric, esophageal, and colon cancers [21,22]. Recent studies have shown that saliva-based biomarkers may reflect systemic inflammatory status and could serve as a promising, easily accessible alternative or complement to blood-based parameters, especially in vulnerable or emergency settings [23,24]. Recent pediatric and translational research has explored salivary inflammatory profiling with encouraging results [25]. However, most available studies were performed on elective surgical cases, leaving the emergency setting largely unexplored, particularly with regard to the prognostic significance of these inflammatory biomarkers in complicated colon cancer.

Therefore, the present study aimed to determine whether routinely available preoperative clinical and systemic inflammatory biomarkers could serve as predictors of postoperative complications (≤30 days), their severity according to the Clavien–Dindo classification, and in-hospital mortality in patients undergoing emergency surgery for colon cancer.

## 2. Materials and Methods

### 2.1. Patients

A retrospective observational study was performed in the General Surgery Department of the Saint Pantelimon Emergency Clinical Hospital, Bucharest, Romania. The study included patients who underwent emergency surgery for colon cancer between October 2021 and November 2025. The research was conducted according to STROBE criteria for cohort studies. The postoperative follow-up period was of 30 days.

Inclusion criteria were: patients aged 18 years and over, that underwent emergency surgery due to one of the following complications of colon cancer: bowel obstruction, colonic perforation, or gastrointestinal bleeding as the initial manifestation.

Exclusion criteria included uncomplicated colon cancer, operated on elective basis, local tumor recurrence, metastatic involvement of the colon from other primary malignancies, death before completion of surgery, pregnancy, and age < 18 years. To avoid the possible bias, patients with co-existing comorbidities previously documented to impact the blood cell-derived indices, such as complicated diabetes, hematological and autoimmune diseases, chronic kidney disease, cirrhosis, associated infections, were excluded. 

### 2.2. Study Outcomes

The primary outcomes were severe postoperative outcomes, described as Clavien-Dindo IIIA or above, documented in the first 30 days after surgery and in-hospital mortality. Secondary outcomes were the rate of anastomotic leaks, the rate of reinterventions, and the length of hospital stay.

The specific aim of the study was to evaluate the correlations between preoperative systemic inflammation biomarkers (NLR, PLR, SII, and CRP) and the postoperative outcomes.

### 2.3. Study Design and Definitions

Patients were divided into two groups: one comprising individuals who developed early postoperative complications (within 30 days or during the same hospital stay) classified as Clavien Dindo grade IIIA or above, and another including patients who experienced an uncomplicated postoperative course.

Demographic data, clinical parameters (e.g., body mass index, type of presentation), laboratory and imaging findings (complete blood count, computed tomography, abdominal ultrasound), surgical parameters (type of intervention, tumor location, presence of perforation, presence of metastases), and postoperative outcomes were extracted from medical records, operative logbooks, and the hospital’s electronic information system.

For each patient, the diagnosis was established based on clinical assessment, imaging evaluation (including abdominal radiography and/or computed tomography), and/or intraoperative findings. Final confirmation of malignancy was obtained by histopathological examination using hematoxylin-eosin staining. Only primary colic adenocarcinomas were included in this study.

All patients underwent a preoperative serological evaluation that included a complete blood cell count, renal and liver function tests, coagulation profile, blood glucose level, and C-reactive protein (CRP). Based on these data, the Neutrophil-to-lymphocyte ratio (NLR), neutrophil-to-monocyte ratio (NMR), platelet-to-lymphocyte ratio (PLR), and the Systemic Immune-Inflammation Index (SII) were calculated. All patients were evaluated by a multidisciplinary team including a general surgeon, anesthesiologist, and a cardiologist or internal medicine specialist. The severity of the comorbidities and perioperative surgical risks was calculated using the Charlson Comorbidity Index (CCI) and the American Society of Anesthesiologists (ASA) score. In cases that met criteria for sepsis or septic shock, diagnosis and treatment followed the guidelines of the Surviving Sepsis Campaign [26].

Surgical interventions were performed within the first 12 h of admission by general surgeons. Informed consent for the appropriate procedure was obtained after a reasonable disclosure [27]. Depending on the patient’s clinical status, tumor location, and surgical risk, the procedures included: diverting colostomies, segmental colectomies, right/left colectomy, or total colectomies with or without primary anastomosis. After surgery, patients were transferred either to the Intensive Care Unit (ICU) for postoperative stabilization or to the general surgery ward, according to adapted protocols [28].

All complications were classified according to the Clavien-Dindo grading system, using data from the hospital’s electronic system. Histopathological assessment was performed on all resected specimens using standard hematoxylin-eosin staining.

### 2.4. Statistical Analysis

Data were analyzed using Microsoft Excel, EasyMedStat (version 3.36; www.easymedstat.com, accessed at 12 December 2025), and Med Calc^®^ Statistical Software (version 22.006, Med Calc Software Ltd., Ostend, Belgium). Normality and hetereoskedasticity of continuous data were assessed with Shapiro-Wilk and Levene’s test, respectively. Continuous variables were presented as mean ± standard deviation (SD) and compared were compared with analysis of variance (ANOVA), Welch’s ANOVA, or Kruskal-Wallis tests according to the data distribution. Discrete outcomes were compared with chi-squared or Fisher’s exact test accordingly. The alpha risk was set to 5% and two-tailed tests were used. Univariable logistic regression analyses were conducted to identify variables associated with postoperative complications and mortality.

The multivariate analysis was performed respecting the Event per Variable (EPV) rule. Taking into account the total number of studied events (severe postoperative complications, in-hospital mortality, and the onset anastomotic leak), a limited number of independent variables was selected based on their strong univariable significance to prevent over-saturation. The predictive performance of significant variables and multivariable models was evaluated using the Receiver Operating Characteristic (ROC) curve analysis. The area under the curve (AUC) was calculated, and optimal cut-off values were determined using the Youden index. A two-tailed *p*-value < 0.05 was considered statistically significant.

#### Sample Size and Power Analysis

A post-hoc power analysis was conducted to validate the adequacy of the sample size for the primary outcome (severe postoperative complications). For an expected Area under the Curve (AUC) of 0.75, a significance level α of 0.05, and a power (1-β) of 0.80, with an allocation ratio of approximately 3:1 between the uncomplicated and complicated groups, the minimum required sample size was calculated to be 148 patients. Our study included 219 patients, which exceeds this requirement and provides a statistical power of 91.2% to detect the observed differences.

**Figure 1:**
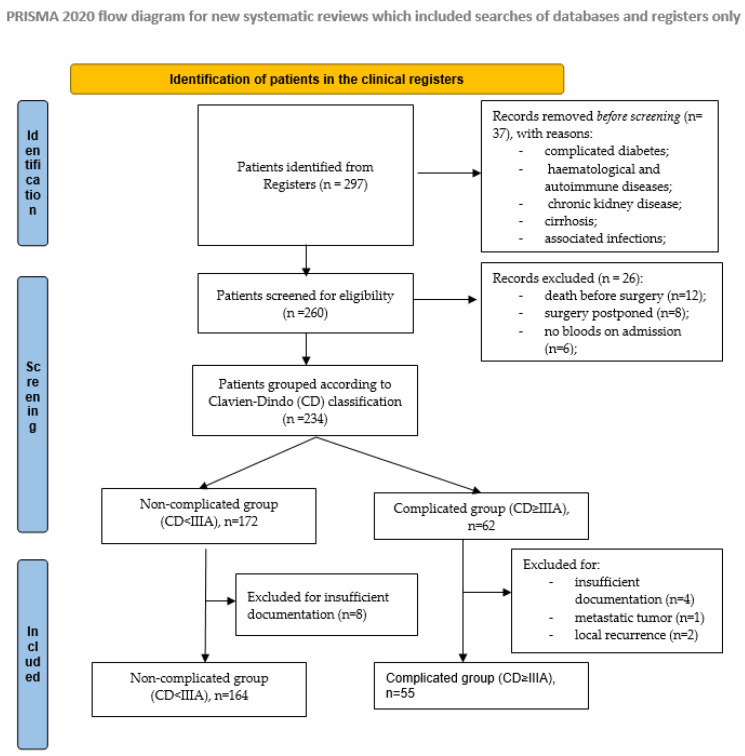
Flowchart for the selection of the patients included in the study group.

In our multivariate analysis, we included the most significant variables identified in the univariate analysis, in a stepwise approach. This “pre-filtering” reduces noise and prevent the risk of overfitting. Furthermore, the Event per Variable (EPV) rule of 10:1 was respected in selecting the appropriate number of variables included in the predictive model. Figure 1.

## 3. Results

### 3.1. Preoperative Data of the Patients Included in the Study Group

After applying the inclusion and exclusion criteria, a total of 219 patients were included in the study, with a mean age of 69.63 (±11.54) years. Demographic and preoperative data are presented in Table 1 and Supplementary file.

The two subgroups were comparable in terms of age and sex distribution. However, patients who developed complications graded as Clavien Dindo ≥ IIIA had significantly higher BMI (29.31 ± 5.69 vs. 27.85 ± 4.49, *p* = 0.007), and CCI scores (7.87 (±1.9) vs. 6.35 (±1.91), *p* < 0.001), as well as increased rates of type II diabetes mellitus.

The need for vasopressor support, the presence of SIRS, and higher ASA score was higher in complicated group (*p* < 0.001, *p* < 0.001, and *p* = 0.024, respectively).

Paraclinical data were consistent with clinical findings, suggesting higher systemic inflammation in the complicated group, with significantly increased values of WBC, CRP, neutrophils, NLR, PLR, NMR, and SII (*p* < 0.001, Table 1). Moreover, the patients that presented postoperative complications associated biological imbalances at admission, with higher levels of urea, creatinine, and blood sugar (*p* < 0.001, Table 1).

### 3.2. Intraoperative Findings and Surgical Approach

Most of the patients included in the study presented tumors located in the left colon (114 cases, 52.05%). The patients that presented postoperative complications ≥ IIIA on Clavien-Dindo grading scale presented a more advanced tumoral stage (*p* = 0.01); with lower grade of differentiation (*p* = 0.005) and advanced loco-regional ganglion invasion (*p* < 0.001) (Table 2).

Tumor resection was performed in most cases, according to the cancer location (Table 2), when technically possible (214 cases, 98.17%). No differences regarding surgical techniques used were found between the 2 study groups (*p* = 0.114%), suggesting that the onset of postoperative adverse outcomes may be correlated with the patients’ biologic status and cancer stage and complications, but not to the surgical technique. However, other relevant intraoperative factors, that could not be evaluated due to the retrospective character of the study, such as the severity of contamination, surgeon experience, and anastomotic technique, may represent important confounders. In the complicated group, the preoperative severe inflammation and the presence of peritonitis were singnificantly higher compared to non-complicated group.

### 3.3. Postoperative Outcomes

In our study, postoperative complications were classified according to the Clavien-Dindo scale. When a patient associated 2 or more complications, the most severe one was used for grading. There was a statistically significant correlation between the length of stay and the mean severity score (*p* = 0.036) (Table 3).

Minor adverse outcomes were noticed in the non-complicated group, graded as I (19 cases, 11.5%), including: superficial surgical site infections (11 cases, 6.7%), seroma (4 cases, 2.4%), prolonged ileus requiring medication (4 cases, 2.4%). Grade II complications were documented in 24 patients (14.6%) in the non-complicated group: urinary infection requiring antibiotics (12 cases, 7.3%), stoma related complications managed conservatory (9 cases; 5.4%), and grade A (mild) anastomotic-leaks, according to ISREC Classification (2010) in 3 cases (1.8%).

In the complicated group, reinterventions were performed for anastomotic leaks (25 cases, 51%), free and contained eviscerations (10 cases, 20.4%), abdominal wall gangrene (3 cases, 6.12%), and stoma related complications (11 cases, 22.44%). Concomitant anastomotic leaks and surgical wound complications were recorded in 18 cases. 25 patients underwent fatal outcome, most of them due to sepsis.

The overall mortality rate in our cohort was 20.54%, with deaths mainly related to septic complications (19 cases, 76%) and, to a lesser extent, to acute cardiac events (6 cases, 24%). The main surgical complication encountered was anastomotic leak (32 cases, 14.6%), out of which 25 cases (78%) required surgical management.

The most reliable serum inflammatory biomarkers associated with adverse outcomes were NLR, PLR, and CRP. Exploratory ROC analyses were used to identify candidate threshold values, and their discriminative performance (AUC ROC), sensitivity, and specificity are presented in Table 4.

### 3.4. Regression Analysis of Preoperative Factors Associated with Adverse Outcomes

A multivariate regression analysis was performed based the the most significant variables, in a stepwise approach. A model including higher values of CCI, CRP, PLR, creatinine, and the association of T2DM was described to be associated with postoperative complications of ≥IIIA, as defined by Clavien-Dindo Classification (Table 5).

**Figure 2:**
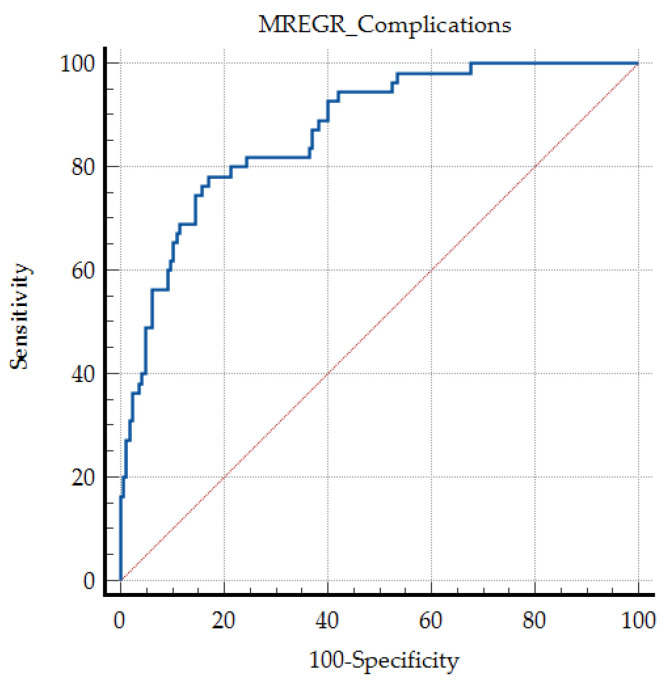
The ROC curve of the described predicting model for complications higher than Clavien Dindo IIIA, for the patients included in the study group (*p* < 0.001); red dotted line—random classifier or random guessing.

The regression model showed a very good discrimination power, with an area under ROC curve (AUC ROC) of 0.870, sensitivity 78.18, and specificity 82.93 (Figure 2).

Furthermore, we carried out a regression analysis for the prediction of anastomotic leak (AL), as an early postoperative complication. We found that higher values of PLR and creatinine, were good predictors for this adverse outcome in T2DM patients (Table 6).

**Figure 3:**
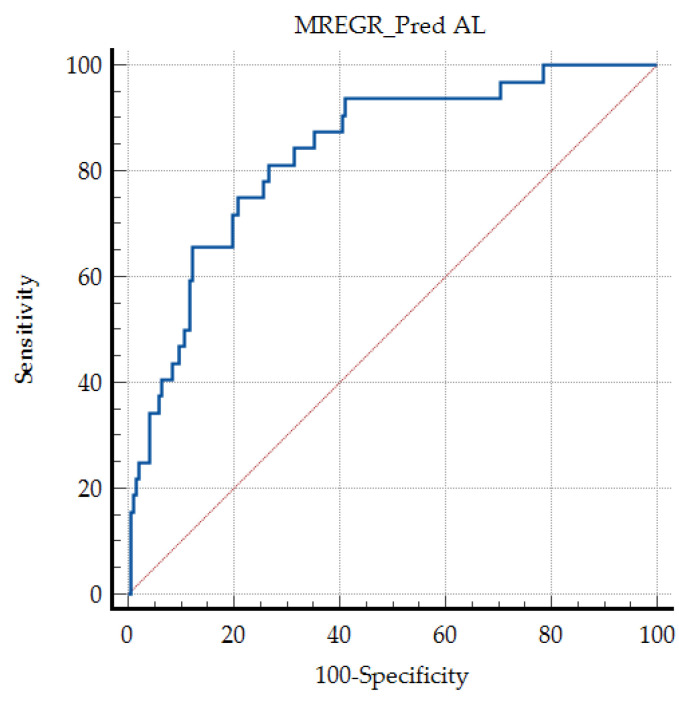
The ROC curve of the described predicting model for anastomotic leaks, for the patients included in the study group (*p* < 0.001); red dotted line—random classifier or random guessing.

Not surprisingly, these factors describe the micro-circulatory and metabolic biological status, that may be associated with vascular impairment at the level of the colic anastomosis. The “Events Per Variable” (EPV) rule remains relevant, the multivariate model is statistically stable and the risk of over fitting is low. The prediction value is very good, with an AUC ROC of 0.834, sensitivity of 81.25%, and specificity of 73.26% (Figure 3).

Regarding fatal outcome, statistical analysis found that the level of preoperative NLR and CRP, as well as the association of peritonitis at admission are strong predictors for death during the hospitalization period, with AUC ROC of 0.891, a sensitivity of 96%, and a specificity of 81.96%. However, due to the limited number of cases (25 patients) in the study group, there may be a medium risk of overfitting. Further studies are needed to validate the results. These findings underline the role of sepsis and systemic inflammation, which was associated with 76% of cases of death (Table 7).

## 4. Discussion

Despite the recent advances in colorectal cancer screening and early detection, about 20% of cases are first diagnosed in emergency, due to a complication requiring admission into a surgical unit. These cases are extremely challenging, requiring a multidisciplinary approach. Emergency colorectal interventions are usually performed in a variable and dynamic context, frequently marked by severe inflammation, metabolic imbalance, and multiple organ dysfunctions [29,30,31]. The specific pathological changes are associated with a 3 to 10 times higher mortality in these cases. Recent studies found that, while age per se is not a contraindication for resection surgery in emergency, the presence of clinical perforation and severe contamination were associated with septic shock and higher mortality in older patients [11,32,33,34]. Emergency colorectal surgery is associated with high 30 days mortality, varying between 6.7 and 37.5% in particularly among frail patients and those presenting with advanced disease or septic complication, and increased morbidity rates, ranging from 33% to 66% [32,33,34,35]. In the present study, the overall in-hospital mortality was of 11.4%, and was mostly caused by uncontrollable preoperative sepsis. Our multivariable model demonstrated excellent discriminative performance for predicting postoperative mortality, achieving an AUCROC of 0.891, a sensitivity of 96%, and a specificity of 81.96%. However, due to the limited number of cases that experienced fatal outcome (25 patients), a moderate risk of over fitting needs to be acknowledged.

Out of the total number of patients included in the study, 25.1% experienced one of more postoperative complications classified as Clavien Dindo IIIA or higher. The most frequent reported adverse events were anastomotic leaks (14.6%) and surgical site infections, either superficial, or deep, both a significant cause of patient sufferance and prolonged hospitalization (11.4%). Early risk stratification and sustained antibiotherapy, hidroelectrolitic imbalance correction and vassopresor medication if required, in a multidisciplinary approach could improve postoperative outcomes [34]. Consequently, preoperative biomarkers have become increasingly used as potential tools for early risk estimation, but their applicability remains poorly defined for emergency colorectal cancer surgery.

This study investigated the value of the clinical and systemic inflammatory indices and in the presence of sepsis, obstruction, or perforation due to colic malignancies. Interpretation of these markers requires not only statistical evaluation, but also an understanding of the pathophysiological processes that determine postoperative morbidity and mortality. A better understanding of their prognostic value for the postoperative outcome could help the clinicians tailor their approach on a personalized manner, thus optimizing the perioperative care. In the present study, we found that higher NLR, PLR and CRP at admission could be useful biomarkers in risk evaluation and perioperative management in patients that required emergency colorectal surgery. While age and tumoral stage were not significant in overall postoperative 30-days mortality and morbidity, a threshold value of NLR > 6.89 was associated with a higher incidence of Clavien–Dindo complications graded as IIIA or higher while preoperative NLR > 9.2 was correlated with fatal outcome.

In our cohort, the mean preoperative CRP level exceeded 90 mg/dL, indicating a strong proinflammatory response, probably driven by significant bacterial contamination associated with circulating malignant cells [36]. CRP demonstrated high discriminative performance in exploratory analyses, with a candidate threshold around 62.8 mg/dL distinguishing patients with Clavien–Dindo ≥ IIIA complications. Moreover, higher values, exceeding 130 mg/dL were associated with fatal outcome (AUC ROC of 0.849). However, all threshold values derived from ROC analyses were exploratory and data-driven, intended for hypothesis generation rather than for defining definitive clinical decision cut-offs.

PLR is a systemic biomarker that characterizes both inflammation and the fluido-coagulation imbalance at the microvascular level. Patients who developed postoperative complications graded Clavien-Dindo ≥ IIIA, presented significantly higher PLR values (512.55 ± 422.42 vs. 260.2 ± 179.17, *p* < 0.001) compared to those who had an uncomplicated postoperative course. Higher PLR values > 334 were associated with overall with Clavien Dindo complications graded as IIIA or higher, and particularly with the risk of developing anastomotic leaks.

However, the individual specificity and sensitivity of each biomarker was moderate in predicting postoperative outcomes, and clinical significance could be improved in association with other variables, such as CCI, the presence of diabetes, and chronic renal disease, in multivariate models. Clinical presentation significantly influenced the risk and the severity of postoperative complications in our study. Peritonitis was more common in patients with adverse postoperative outcomes (OR = 4.46, 95% CI: 2.02–9.86) and is a strong predictor of fatal outcome. Clavien, (OR = 9.96; 95% CI: 2.74–36.1). These findings may be multifactorial. Most patients with peritoneal contamination required vasopressor agents for adequate tissue perfusion, had organ failures, or severe systemic inflammation, factors recognized for poorer postoperative outcomes in colon cancer [36,37].

The management of complicated colorectal cancer includes decisions about the appropriate strategy: tumor resection, diversions, ostomy, or minimal procedures, known as “bridge to surgery” in obstructive disease [37,38,39]. Colorectal anastomotic leaks may be regarded as a process of failed wound healing, for which both preexisting comorbidities and preoperative systemic inflammation evaluation might be a promising research tool to decrease leaks rates [37]. Implementing multivariate prediction models could identify the “high risk” anastomoses, and may help the surgeon to choose a personalized therapeutic approach. Moreover, high CRP and NLR values in a patient admitted with peritonitis due to perforated colon cancer could be an indicator of high mortality risk. Perioperative antibiotic and supportive therapy and minimal surgery aiming infection control may prevent fatal outcome.

CCI is a widely useful tool in the preoperative evaluation of patients with colon and gastric cancer. Previous studies concluded that a CCI score above 4 is associated with a 3.5-fold increased risk of postoperative complications [36,40,41]. Zhang et al. [41] found that CCI > 3 correlates well with higher health expenses; prolonged hospital stays and in hospital morbidity in patients with colorectal cancer. In this study, we found that CCI was an independent factor for complications graded as IIIA or more on Clavien-Dindo Classification, together with higher PLR, CRP, serum creatinine, and the presence of T2DM.

In our study, both the presence of diabetes, as well as higher values of blood sugar were strongly associated with adverse outcomes. Moreover, patients with diabetes mellitus had an approximately a fivefold increased risk of developing postoperative complications (OR = 5.5, 95%, *p* < 0.001). Several studies confirmed higher rates of complications in diabetic patients may be explained by defective immune mechanisms, such as opsonization, phagocytosis, chemotaxis, and neutrophil adherence [41,42,43], but also due to changes in microvasculature and detailed wound repair. Tan et al. [43], in a meta-analysis of 93,173 diabetic patients undergoing colorectal surgery, reported significantly higher rates of anastomotic leakage, surgical wound infections, acute kidney injury, as well as increased mortality. These vulnerable patients would benefit of an enhanced recovery program (ERP) to reduce surgical stress and its metabolic consequences [41], however implementing this strategy is challenging in emergency surgery. The explanation for the higher rates of complications in our cohort is the emergency presentation of patients [5], the presence of organ dysfunctions, the need for vasopressor agents, or other associated comorbidities [44].

Systemic inflammation plays a central role in the initiation, progression, and clinical course of colon cancer. Proinflammatory cytokines, such as IL-6 and TNF-α, activate JAK/STAT and nuclear factor κB signaling pathways, thereby promoting the proliferation and migration of the malignant cells, as well as angiogenesis [45]. Due to this inflammatory response, several serological parameters such as CRP [46], number of WBC, neutrophil and platelet-related indices, can be significantly altered [47]. In clinical practice, such changes may be caused by the presence of malignant cell in the blood stream, or may occur secondary to common complications in advanced colon cancer (including bowel obstruction, perforation, or systemic inflammatory responses associated with tumor progression) [48]. 

CRP is one of the most studied inflammatory markers. It is a liver-synthesized acute-phase reactant whose synthesis is induced by elevated levels of IL-6 in response to trauma, infection, or ischemia [49]. In healthy individuals, baseline hepatic production is approximately 2.4 mg/day. In the presence of an inflammatory stimulus, CRP synthesis begins within 6–12 h and typically peaks between 24–72 h, with serum levels increasing up to 1000-fold. In conditions of severe systemic inflammation, CRP production can reach approximately 174 mg/day [49,50,51].

The pathophysiological mechanism between postoperative complications and systemic inflammation is likely related to the immune dysfunction present in sepsis [52]. Bacterial lipopolysaccharides and cellular debris are present in large quantities and will cause persistent antigenic stimulation and an increase in IL-6 and CRP [53]. This pro-inflammatory environment activates tissue factor and plasminogen activator inhibitor-1, promoting microvascular thrombosis, which may contribute to higher rates of anastomotic leakage, delayed healing, and increased susceptibility to bacterial infections [54].

NLR is an inflammatory marker that reflects the balance between innate (neutrophils) and adaptive (lymphocytes) immunity [55]. Elevated neutrophil counts are associated with pro-metastatic and tumor-promoting mechanisms. They are responsible for increased production of reactive oxygen species, genomic instability, and enhanced angiogenesis [56]. In contrast, lymphocytes—especially T-cells—are involved in antitumor responses [57]. An increased NLR is a marker of poor oncological prognosis [58].

In our study, patients who developed surgical complications exhibited a significantly higher NLR compared to those who experienced an uncomplicated postoperative course (13.81 ± 10.51 vs. 6.73 ± 6.43, *p* < 0.001). In a multivariate analysis, NLR and CRP were strong predictors for fatal outcome, in patients with peritonitis at admission (AUC ROC 0.891, a sensitivity of 96%, and a specificity of 81.96%). However, high PLR were better correlated with overall complications, and with anastomotic leaks.

Elevated platelet counts have been associated with adverse clinical outcomes in colon cancer [59]. Tumor cells can directly activate circulating platelets, promoting the release of inflammatory and prothrombotic mediators that disrupt intercellular junctions and alter surface adhesion molecules, thereby increasing the potential for both local invasion and distant metastasis [60]. Through these mechanisms, an elevated PLR may be associated with increased postoperative morbidity [61]. The pathophysiological rationale for these findings may be related to the cancer-associated hypercoagulable state, which promotes microthrombosis, delayed tissue healing, and low immunologic reactivity [62]. Compared with previous studies evaluating the prognostic value of PLR in colon and rectum cancer [63,64], PLR levels observed in our cohort were significantly higher, most likely reflecting the greater inflammatory burden and disease severity in complicated emergency cases, which are known to be associated with poorer postoperative outcomes. 

Colorectal cancer patients that undergo surgery, particularly on emergency basis, are at high risk of developing SSI, a major cause of postoperative morbidity, increased hospital stay, and healthcare associated costs. Higher TNM stages were associated with higher SSI rate [15,65,66]. Butyrylcholinesterase (BChE), a non-specific cholinesterase enzyme, has been correlated with the risk of hepatic dysfunction progression and, more recently, to multiple conditions associated with systemic inflammation. Recent studies found low levels of Butyrylcholinesterase in the first and third post-surgery to be an important biomarker correlated with postoperative SSI in colorectal cancer surgery, but not with sepsis [67].

Several scoring systems were investigated for their predictive value in predicting colorectal cancer complication in the early postoperative period. APACHE II (Acute Physiology and Chronic Health Evaluation II) is a severity-of-disease scoring system used mainly in intensive care units (ICUs) to estimate the risk of mortality in critically ill adults. It requires a minutious preoperative evaluation, taking into account multiple parameters such as blood pressure, blood pressure, heart rate, respiratory rate, blood chemistry, patient age, and chronic health conditions. While its preoperative value was well documented to be a useful tool in predicting outcomes, serial postoperative measures are not so clinical meaningful [68]. Complimentary, NEWS 2 (National Early Warning Score 2) is a track-and-trigger early warning score designed to detect clinical deterioration early in hospitalized patients, based on 7 physiological measurements routinely recorded at the bedside (respiratory rate, oxygen saturation, blood pressure, heart rate, temperature, level of consciousness, and the need for oxygen supplementation). Recent studies found that combining the 2-evaluation system could be valuable in the monitoring of complications during the postoperative period of colorectal surgical patients [68,69].

In emergency surgery, acute kidney injury (AKI) is a common and severe postoperative condition, often linked to hemodynamic instability and systemic inflammation. Its occurrence is associated with an increased risk of postoperative morbidity and higher mortality rates [70]. Consequently, preoperative assessment of renal function is essential, as it provides valuable prognostic information regarding the likelihood and severity of postoperative complications. In our cohort, we describe a multivariate model, including serum creatinine, T2DM and PLR with a very good predictive value for AL (AUC ROC of 0.834, sensitivity of 81.25%, and specificity of 73.26%). Patients with impaired renal function, especially those with an estimated creatinine clearance below 70 mL/min, exhibit an exaggerated systemic inflammatory response due to membranous dysfunction and altered immune signaling, making them more susceptible to both infectious and non-infectious postoperative complications [71,72]. 

This study has several limitations. The present study has a retrospective, single-center design conducted in a predominantly urban population; therefore, the findings cannot be generalized to the entire regional population. One limitation is the lack of external validation for the proposed predictive models. Further multicentric studies are necessary for clinical validation of the results. In the present study, the main reported outcome is the rate on 30-days postoperative complications. Other limitation is the absence of long-term oncologic outcomes. Although the surgical teams are experienced and a variety of surgical procedures can be performed, the colorectal procedures are not exclusively their area of specialization, which may introduce variability in operative techniques and postoperative outcomes. In addition, accurate documentation of postoperative complications is inherently difficult, as adverse events may be underreported due to the psychological burden placed on surgical teams or concerns about potential medico-legal implications [73,74]. The exploratory nature of the ROC-derived thresholds and the lack of external validation represent important limitations. Importantly, the threshold values reported in this study should be interpreted as exploratory. They were derived from a single-center retrospective cohort and reflect data-driven optimization rather than externally validated clinical cutoffs. As such, these values are best viewed as hypothesis-generating and require confirmation in independent and prospective cohorts before clinical implementation.

Future studies, involving multiple centers and larger cohort of patients are necessary to validate the results in clinical practice. Additional works are also needed to explore other clinically relevant or serological parameters, including the potential influence of these biomarkers on intraoperative decision-making (e.g., primary anastomosis versus stoma formation). Furthermore, the contribution of intraoperative factors—including tumor location, presence and site of perforation, metastatic spread, and macroscopic characteristics of intraperitoneal fluid—to postoperative mortality and morbidity warrants further dedicated investigations.

## 5. Conclusions

This study highlights the prognostic value of preoperative inflammatory biomarkers and renal function, together with clinical comorbidity scores, in patients undergoing emergency surgery for complicated colon cancer. These findings should not be interpreted as establishing definitive decision thresholds, but rather as exploratory signals that may inform future validation studies and refined risk stratification tools. Elevated CRP, NLR, and PLR were all significantly associated with both the occurrence and severity of postoperative complications. The early onset of the anastomotic leaks, however, were better correlated with higher PLR values and associated co-morbidities, including T2DM and impaired renal function. These results support the use of an integrated risk stratification approach in emergency colorectal cancer surgery, which may facilitate early identification of high-risk patients, guide intraoperative strategies, and allow for individualized perioperative care.

## Figures and Tables

**Table 1:** Clinical and paraclinical characteristics of the patients included in the study group.

Variable	Non-Complicated Group (*n* = 164)	Complicated Group (*n* = 55)	*p*-Value	All Patients (*n* = 219)
Gender (N, %) M	94 (57.32%)	31 (56.36%)	>0.999 ^1^	125 (57.08%)
Age (years, mean ± SD)	68.93 (±11.87)	71.69 (±10.45)	0.104 ^2^	69.63 (±11.54)
BMI (mean ± SD)	27.85 (±4.49)	29.31 (±5.69)	0.007 *^2^	28.22 (±4.83)
CCI (mean ± SD)	6.35 (±1.91)	7.87 (±1.9)	<0.001 *^2^	6.73 (±2.01)
BMI > 30 (N, %)	45 (27.44%)	29 (52.73%)	0.001 *^1^	74 (33.79%)
ASA grade (N, %) 2 3 4 5	18 (10.98%) 86 (52.44%) 58 (35.37%) 2 (1.22%)	0 (0.0%) 15 (27.27%) 38 (69.09%) 2 (3.64%)	<0.001 *^3^	8 (8.22%) 101 (46.12%) 96 (43.84%) 4 (1.83%)
T2DM (N, %)	26 (15.85%)	28 (50.91%)	<0.001 *^1^	54 (24.66%)
Admission (N, %) Occlusion Peritonitis Hemorrhage	96 (58.54%) 18 (10.98%) 50 (30.49%)	25 (45.45%) 24 (43.64%) 6 (10.91%)	<0.001 *^1^	121 (55.25%) 42 (19.18%) 56 (25.57%)
SIRS at admission (N, %)	28 (17.07%)	32 (58.18%)	<0.001 *^1^	60 (27.4%)
Single/Multiple organ failure (N, %)	30 (18.29%)	39 (70.91%)	<0.001 *^1^	69 (31.51%)
Vassopressor use (N, %)	29 (17.68%)	26 (47.27%)	<0.001 *^1^	55 (25.11%)
WBC (mean ± SD)	10.96 (±5.05)	15.09 (±9.0)	0.001 *^2^	11.99 (±6.5)
Monocytes (cells × 10^3^/MMC, mean ± SD)	0.896 (±0.594)	0.856 (±0.449)	0.694 ^2^	0.886 (±0.559)
Neutrophils (cells × 10^3^/MMC, mean ± SD)	8.83 (±5.83)	12.6 (±8.18)	<0.001 *^2^	9.78 (±6.67)
Lymphocytes (cells × 10^3^/MMC, mean ± SD)	1.75 (±1.2)	1.28 (±0.943)	<0.001 *^2^	1.63 (±1.16)
Thrombocytes (cells × 10^3^/MMC, mean ± SD)	348.65 (±136.5)	435.07 (±171.09)	0.001 *^2^	370.36 (±149.97)
Hb (mg/dL, mean ± SD)	11.52 (±3.26)	9.86 (±3.45)	0.002 *^2^	11.1 (±3.37)
Urea (mg/dL, mean ± SD)	50.4 (±34.33)	77.58 (±45.54)	<0.001 *^2^	57.22 (±39.09)
Creatinine (mg/dL, mean ± SD)	1.08 (±0.604)	1.73 (±0.932)	<0.001 *^2^	1.24 (±0.751)
Blood sugar (mg/dL, mean ± SD)	131.8 (±50.83)	160.04 (±65.31)	<0.001 *^2^	138.9 (±55.9)
CRP (mean ± SD)	65.18 (±75.62)	166.19 (±116.81)	<0.001 *^2^	90.55 (±97.67)
SII (mean ± SD)	2256.85 (±2250.16)	6286.79 (±6118.22)	<0.001 *^2^	3268.93 (±4006.59)
NMR (mean ± SD)	12.04 (±9.03)	17.64 (±11.75)	<0.001 *^2^	13.45 (±10.03)
NLR (mean ± SD)	6.73 (±6.43)	13.81 (±10.51)	<0.001 *^2^	8.51 (±8.22)
PLR (mean ± SD)	260.2 (±179.17)	512.55 (±422.42)	<0.001 *^2^	323.58 (±282.61)

Footnote: * statistically significant (*p* < 0.05); ^1^ Chi-squared test; ^2^ Mann-Whitney test; ^3^ Fisher’s exact test; M—males; N—number; SD—standard deviation; BMI—body mass index; CCI—Charlson comorbidity index; ASA—American Society of Anesthesiologists; SIRS—systemic inflammatory response syndrome; T2DM—type 2 diabetes mellitus; CRP—C reactive protein; WBC—white blood cells; Hb—hemoglobin; SII—systemic immune inflammatory index; NLR—neutrophil-to-lymphocyte ratio; PLR—platelet-to lymphocyte ratio; NMR—neutrophil-to-monocyte ratio.

**Table 2:** Intraoperative and histopathological data of the patients included in the study.

Variable	Non-Complicated Group (*n* = 164)	Complicated Group (*n* = 55)	*p*-Value	All Patients (*n* = 219)
**Tumor location** left colon right colon transverse colon	82 (50.0%) 78 (47.56%) 4 (2.44%)	32 (58.18%) 21 (38.18%) 2 (3.64%)	0.455 ^1^	114 (52.05%) 99 (45.21%) 6 (2.74%)
T Staging T2 T3 T4	7 (4.27%) 77 (46.95%) 80 (48.78%)	0 (0.0%) 16 (29.09%) 39 (70.91%)	0.01 *^2^	7 (3.2%) 93 (42.47%) 119 (54.34%)
N Staging N0 N1 N2 N3	65 (39.63%) 54 (32.93%) 42 (25.61%) 3 (1.83%)	11 (20.0%) 14 (25.45%) 25 (45.45%) 5 (9.09%)	<0.001 *^2^	76 (34.7%) 68 (31.05%) 67 (30.59%) 8 (3.65%)
Grade of differentiation G1 G2 G3	54 (32.93%) 73 (44.51%) 37 (22.56%)	6 (10.91%) 30 (54.55%) 19 (34.55%)	0.005 *^2^	60 (27.4%) 103 (47.03%) 56 (25.57%)
Peritoneal carcinomatosis	33 (20.12%)	16 (29.09%)	0.232 ^2^	49 (22.37%)
Vascular invasion	133 (81.1%)	50 (90.91%)	0.236 ^2^	183 (83.56%)
Neural invasion	129 (78.66%)	50 (90.91%)	0.067 ^2^	179 (81.74%)
Type of surgical resection Right/extended right HC Total colectomy Left/extended left HC Non resectable	76 (46.34%) 33 (20.12%) 53 (32.32%) 2 (1.22%)	22 (40.0%) 18 (32.73%) 13 (23.64%) 2 (3.64%)	0.114 ^1^	98 (44.75%) 51 (23.29%) 66 (30.14%) 4 (1.83%)
Colostomy/Ileostomy	51 (31.1%)	25 (45.45%)	0.076	76 (34.7%)

Footnote: * statistically significant (*p* value < 0.05); ^1^ Fisher’s exact test; ^2^ Chi-squared test; T—tumor; N—adenopathy; HC—hemicolectomy.

**Table 3:** Postoperative outcomes in the study groups.

Variable	Non-Complicated Group (*n* = 164)	Complicated Group (*n* = 55)	*p*-Value	All Patients (*n* = 219)
Hospital stay (days, mean ± SD)	9.41 (±5.57)	13.33 (±9.85)	0.036 *	10.39 (±7.07)
Clavien-Dindo staging of complications: I (minor) II (requiring pharmacological treatment) III (requiring surgical/interventional therapy) IV (requiring intensive care) V (death)	19(11.5%) 24 (14.6%) 0 (0%) 0 (0%) 0 (0%)	0 0 13 (23.6%) 17 (30.9%) 25 (45.45%)	<0.001 *	19 (8.6%) 24 (10.9%) 13 (5.9%) 17 (7.76%) 25 (20.54%)
Reinterventions	0 (0%)	49 (89.09%)	<0.001 *	49 (22.3%)

Footnote: SD—standard deviation; *—statistically significant (*p* value < 0.05).

**Table 4:** Exploratory threshold values of NLR, PLR and CRP for different types of postoperative adverse outcomes.

Type of Complications	NLR	PLR	CRP
Complications CD ≥ IIIA; Sensitivity Specificity Exploratory threshold value AUC ROC	72.7% 75.6% >6.89 0.748	69.1% 77.4% >334.2 0.726	85.5% 69.5% >62.8 0.799
Anastomotic leaks: Sensitivity Specificity Exploratory threshold value AUC ROC	78.1% 71.1% >7.35 0.743	75% 72.7% >334.2 0.743	75% 70% >81.4 0.712
Fatal outcome: Sensitivity Specificity Exploratory threshold value AUC ROC	76% 78.3% >9.2 0.807	64% 79.4% >391 0.702	76% 83% >130 0.849

Footnote: AUC ROC—area under Receive Operating Curve; CD—Clavien Dindo severity scale; NLR—neutrophil-to-lymphocyte ratio; PLR—platelet-to lymphocyte ratio; CRP—C reactive protein.

**Table 5:** Regression model predicting postoperative Clavien-Dindo complications ≥ IIIA.

Independent Variables	Coefficient	Std. Error	95% CI	t	*p*	r_partial_	r_semipartial_	VIF
(Constant)	−0.3543	0.08884	−0.5294 to −0.1792	−3.9880	0.0001			
CCI	0.03507	0.01258	0.01028 to 0.05987	2.7888	0.0058	0.1877	0.1528	1.135
PLR	0.0002943	0.00009289	0.0001112 to 0.0004774	3.1687	0.0018	0.2122	0.1736	1.221
Creatinine	0.1153	0.03435	0.04762 to 0.1831	3.3575	0.0009	0.2242	0.1839	1.179
CRP	0.0009704	0.0002836	0.0004114 to 0.001529	3.4218	0.0007	0.2283	0.1874	1.359
T2DM	0.1732	0.05983	0.05522 to 0.2911	2.8941	0.0042	0.1945	0.1585	1.178

Footnote: Std. Error—standard error; CI—confidence interval; t—t statistic; *p* value—statistically significant at <0.05; r _partial_—partial correlation; r s_emipartial_—semipartial correlation; VIF—Variance Inflation Factor; CCI—Charlson comorbidity index; PLR—platelet-to lymphocyte ratio; CRP—C reactive protein; T2DM—type 2 diabetes mellitus.

**Table 6:** Regression analysis for predicting anastomotic leaks (AL).

Independent Variables	Coefficient	Std. Error	95% CI	t	*p*	r_partial_	r_semipartial_	VIF
(Constant)	−0.1206	0.04506	−0.2094 to −0.03177	−2.6762	0.0080			
Creatinine	0.1277	0.02916	0.07021 to 0.1852	4.3788	<0.0001	0.2861	0.2649	1.050
T2DM	0.2155	0.05163	0.1137 to 0.3172	4.1733	<0.0001	0.2737	0.2525	1.084
PLR	0.0001689	0.00007986	0.00001145 to 0.0003263	2.1144	0.0356	0.1427	0.1279	1.116

Footnote: Std. Error—standard error; CI—confidence interval; r _partial_—partial correlation; r _semipartial_—semipartial correlation; VIF—Variance Inflation Factor; PLR—platelet-to lymphocyte ratio; T2DM—type 2 diabetes mellitus.

**Table 7:** Regression analysis for fatal outcome.

Independent Variables	Coefficient	Std. Error	95% CI	T	*p*	r_partial_	r_semipartial_	VIF
(Constant)	−0.06374	0.02850	−0.1199 to −0.007563	−2.2364	0.0264			
NLR	0.01014	0.002469	0.005274 to 0.01501	4.1070	0.0001	0.2697	0.2363	1.229
CRP	0.0006871	0.0002608	0.0001731 to 0.001201	2.6348	0.0090	0.1769	0.1516	1.937
Peritonitis	0.1611	0.06349	0.03595 to 0.2862	2.5372	0.0119	0.1705	0.1460	1.797

Footnote: Std. Error—standard error; CI—confidence interval; r _partial_—partial correlation; r _semipartial_—semipartial correlation; VIF—Variance Inflation Factor; NLR—neutrophil-to lymphocyte ratio; CRP—C reactive protein.

## Data Availability

Data available on request.

## Supplementary Materials

The following supporting information can be downloaded at: https://www.mdpi.com/article/10.3390/jcm15041627/s1, Supplementary file: Anonymized database.

## References

[1] Roshandel G., Ghasemi-Kebria F., Malekzadeh R. (2024). Colorectal Cancer: Epidemiology, Risk Factors, and Prevention. Cancers.

[2] Verkuijl S.J., Jonker J.E., Trzpis M., Burgerhof J.G.M., Broens P.M.A., Furnée E.J.B. (2021). Functional outcomes of surgery for colon cancer: A systematic review and meta-analysis. Eur. J. Surg. Oncol..

[3] Zheng S., Schrijvers J.J., Greuter M.J., Kats-Ugurlu G., Lu W., de Bock G.H. (2023). Effectiveness of Colorectal Cancer (CRC) Screening on All-Cause and CRC-Specific Mortality Reduction: A Systematic Review and Meta-Analysis. Cancers.

[4] Siegel R.L., Miller K.D., Wagle N.S., Jemal A. (2023). Cancer statistics, 2023. CA Cancer J. Clin..

[5] Golder A.M., McMillan D.C., Horgan P.G., Roxburgh C.S. (2022). Determinants of emergency presentation in patients with colorectal cancer: A systematic review and meta-analysis. Sci. Rep..

[6] Dahdaleh F.S., Sherman S.K., Poli E.C., Vigneswaran J., Polite B.N., Sharma M.R., Catenacci D.V., Maron S.B., Turaga K.K. (2018). Obstruction predicts worse long-term outcomes in stage III colon cancer: A secondary analysis of the N0147 trial. Surgery.

[7] Goldsbury D.E., Feletto E., Weber M.F., Haywood P., Pearce A., Lew J.B., Worthington J., He E., Steinberg J., O’Connell D.L. (2021). Health system costs and days in hospital for colorectal cancer patients in New South Wales, Australia. PLoS ONE.

[8] Zhang J., Zhu H., Yang W., Liu X., Zhang D., Jiang X., Yang L., Zhou Z. (2022). Endoscopic stent versus diverting stoma as a bridge to surgery for obstructive colorectal cancer: A systematic review and meta-analysis. Langenbeck’s Arch. Surg..

[9] Faes S., Hübner M., Girardin T., Demartines N., Hahnloser D. (2021). Rate of stoma formation following damage-control surgery for severe intra-abdominal sepsis: A single-centre consecutive case series. BJS Open.

[10] Alverdy J.C., Schardey H.M. (2021). Anastomotic Leak: Toward an Understanding of Its Root Causes. J. Gastrointest. Surg..

[11] Raab S., Wingelmayr O., Shamiyeh A. (2025). Management of complications in colorectal surgery. Eur. Surg..

[12] Andras D., Lazar A.M., Crețoiu D., Berghea F., Georgescu D.E., Grigorean V., Iacoban S.R., Mastalier B. (2024). Analyzing postoperative complications in colorectal cancer surgery: A systematic review enhanced by artificial intelligence. Front. Surg..

[13] Wang Y., Li X., Yu Y., Liang J. (2023). Risk factors for sepsis in patients with colorectal cancer complicated with gastrointestinal perforation and its impact on prognosis. J. Gastrointest. Oncol..

[14] Panos G., Mulita F., Akinosoglou K., Liolis E., Kaplanis C., Tchabashvili L., Vailas M., Maroulis I. (2021). Risk of surgical site infections after colorectal surgery and the most frequent pathogens isolated: A prospective single-centre observational study. Med. Off. Publ. Med. Assoc. Zenica-Doboj Canton Bosnia Herzeg..

[15] Cunha T., Miguel S., Maciel J., Zagalo C., Alves P. (2025). Surgical site infection prevention care bundles in colorectal surgery: A scoping review. J. Hosp. Infect..

[16] Bu N., Liu C., Kong Z., Gao W., Zhu Y. (2025). Predictive value of preoperative inflammatory response markers on short-term postoperative complications following colorectal surgery: A secondary analysis of a randomized clinical trial. Front. Med..

[17] Aoyama T., Yukawa N., Saito A. (2024). Clinical Impact of Nutrition and Inflammation Assessment Tools in Colorectal Cancer Treatment. Anticancer. Res..

[18] Goodwin A.M., Kurapaty S.S., Inglis J.E., Divi S.N., Patel A.A., Hsu W.K. (2024). A meta-analysis of the American college of surgeons risk calculator’s predictive accuracy among different surgical sub-specialties. Surg. Pract. Sci..

[19] Koedam T.W.A., Bootsma B.T., Deijen C.L., van de Brug T., Kazemier G., Cuesta M.A., Fürst A., Lacy A.M., Haglind E., Tuynman J.B. (2022). Oncological Outcomes After Anastomotic Leakage After Surgery for Colon or Rectal Cancer: Increased Risk of Local Recurrence. Ann. Surg..

[20] Warps A.K., Tollenaar R.a.E.M., Tanis P.J., Dekker J.W.T., Audit D.C. (2022). Postoperative complications after colorectal cancer surgery and the association with long-term survival. Eur. J. Surg. Oncol..

[21] Menyhart O., Fekete J.T., Győrffy B. (2024). Inflammation and Colorectal Cancer: A Meta-Analysis of the Prognostic Significance of the Systemic Immune–Inflammation Index (SII) and the Systemic Inflammation Response Index (SIRI). Int. J. Mol. Sci..

[22] Ming-Sheng F., Mei-Ling D., Xun-Quan C., Yuan-Xin H., Wei-Jie Z., Qin-Cong P. (2022). Preoperative Neutrophil-to-Lymphocyte Ratio, Platelet-to-Lymphocyte Ratio, and CEA as the Potential Prognostic Biomarkers for Colorectal Cancer. Can. J. Gastroenterol. Hepatol..

[23] Milunović K.P., Stanišić L., Barić T., Meštrović J., Todorić D., Domić D.Š., Jerončić A., Pogorelić Z. (2025). Salivary C-Reactive Protein: A Non-Invasive Alternative to Serum CRP in Pediatric Acute Appendicitis. Molecules.

[24] Arbildo-Vega H.I., Panda S., Cruzado-Oliva F.H., Vásquez-Rodrigo H., Aguirre-Ipenza R., Meza-Málaga J.M., Luján-Valencia S.A., Luján-Urviola E., Farje-Gallardo C.A., Castillo-Cornock T.B. (2025). Salivary biomarkers for the prognosis of oncological and infectious diseases: A systematic review. Front. Dent. Med..

[25] Pogorelić Z., Jukić M., Žuvela T., Milunović K.P., Maleš I., Lovrinčević I., Kraljević J. (2025). Salivary Biomarkers in Pediatric Acute Appendicitis: Current Evidence and Future Directions. Children.

[26] Evans L., Rhodes A., Alhazzani W., Antonelli M., Coopersmith C.M., French C., Machado F.R., Mcintyre L., Ostermann M., Prescott H.C. (2021). Surviving Sepsis Campaign: International Guidelines for Management of Sepsis and Septic Shock 2021. Crit. Care Med..

[27] Serban D., Spataru R.I., Vancea G., Balasescu S.A., Socea B., Tudor C., Dascalu A.M. (2020). Informed consent in all surgical specialties: From legal obligation to patient satisfaction. Rom J Leg Med.

[28] Nates J.L., Nunnally M., Kleinpell R., Blosser S., Goldner J., Birriel B., Fowler C.S., Byrum D., Miles W.S., Bailey H. (2016). ICU Admission, Discharge, and Triage Guidelines: A Framework to Enhance Clinical Operations, Development of Institutional Policies, and Further Research. Crit. Care Med..

[29] Tebala G.D., Natili A., Gallucci A., Brachini G., Khan A.Q., Tebala D., Mingoli A. (2018). Emergency treatment of complicated colorectal cancer. Cancer Manag. Res..

[30] Savlovschi C., Serban D., Trotea T., Borcan R., Dumitrescu D. (2013). Post-surgery morbidity and mortality in colorectal cancer in elderly subjects. Chirurgia.

[31] Savlovschi C., Comandaşu M., Şerban D. (2013). Particularities of Diagnosis and Treatment in Synchronous Colorectal Cancers (SCC). Chirurgia.

[32] Le H.D., Wolinska J.M., Baertschiger R.M., Himidan S.A. (2023). Complication Is Inevitable, but Suffering is Optional-Psychological Aspects of Dealing with Complications in Surgery. Eur. J. Pediatr. Surg..

[33] Doran H., Pătraşcu T., Catrina E., Mihalache O. (2008). Hartmann ‘s procedure. A 30 years one-centre clinical experience. Chirurgia.

[34] Krutsri C., Sumpritpradit P., Singhatas P., Thampongsa T., Phuwapraisirisan S., Gesprasert G., Jirasiritham J., Choikrua P. (2021). Morbidity, mortality, and risk factors of emergency colorectal surgery among older patients in the Acute Care Surgery service: A retrospective study. Ann. Med. Surg..

[35] Kedareswar, Abhinaya R.P., Nr V.P., Sr A., M R., Jain A., K G., Kayal S. (2026). Comparative Outcomes of Colorectal Cancer Patients Undergoing Elective and Emergency Surgeries: A Propensity Score Matched Cohort Study. J. Gastrointest. Cancer.

[36] Bhattacharjee H.K., Kaviyarasan Mp Singh K.h.J., Don Jose K., Suhani S., Joshi M., Parshad R. (2023). Age adjusted Charlson comorbidity index (a-CCI) AS a tool to predict 30-day post-operative outcome in general surgery patients. ANZ J. Surg..

[37] Holmgren K., Jonsson P., Lundin C., Matthiessen P., Rutegård J., Sund M., Rutegård M. (2022). Preoperative biomarkers related to inflammation may identify high-risk anastomoses in colorectal cancer surgery: Explorative study. BJS Open.

[38] Guo J., Chok A.Y., Lim H.J., Tay W.X., Lye W.K., Samarakoon L.B., Tan E.J., Mathew R. (2021). Prognostic Value of Neutrophil-to-Lymphocyte Ratio in Obstructing Colorectal Cancer Treated by Endoscopic Stenting as a Bridge to Surgery. Ann. Coloproctology.

[39] DESimone B., Abu-Zidan F.M., Podda M., Pellino G., Sartelli M., Coccolini F., DISaverio S., Biffl W.L., Kaafarani H.M., Moore E.E. (2024). The management of complicated colorectal cancer in older patients in a global perspective after COVID-19: The CO-OLDER WSES project. Minerva Surg..

[40] Renzi C., Lyratzopoulos G., Hamilton W., Maringe C., Rachet B. (2019). Contrasting effects of comorbidities on emergency colon cancer diagnosis: A longitudinal data-linkage study in England. BMC Health Serv. Res..

[41] Zhang X., Wang X., Wang M., Gu J., Guo H., Yang Y., Liu J., Li Q. (2023). Effect of comorbidity assessed by the Charlson Comorbidity Index on the length of stay, costs, and mortality among colorectal cancer patients undergoing colorectal surgery. Curr. Med. Res. Opin..

[42] Wankhede D., Halama N., Kloor M., Brenner H., Hoffmeister M. (2025). Diabetes and Colorectal Cancer Risk and Survival According to Tumor Immunity Status. J. Clin. Oncol. Off. J. Am. Soc. Clin. Oncol..

[43] Tan D.J.H., Yaow C.Y.L., Mok H.T., Ng C.H., Tai C.H., Tham H.Y., Foo F.J., Chong C.S. (2021). The influence of diabetes on postoperative complications following colorectal surgery. Tech. Coloproctology.

[44] Bawa D., Khalifa Y.M., Khan S., Norah W., Noman N. (2023). Surgical outcomes and prognostic factors associated with emergency left colonic surgery. Ann. Saudi Med..

[45] Zamaray B., van Velzen R.A., Snaebjornsson P., Consten E.C.J., Tanis P.J., van Westreenen H.L. (2023). Outcomes of patients with perforated colon cancer: A systematic review. Eur. J. Surg. Oncol..

[46] Cheng E., Shi Q., Shields A.F., Nixon A.B., Shergill A.P., Ma C., Guthrie K.A., Couture F., Kuebler P., Kumar P. (2023). Association of Inflammatory Biomarkers With Survival Among Patients With Stage III Colon Cancer. JAMA Oncol..

[47] Rhodes B., Fürnrohr B.G., Vyse T.J. (2011). C-reactive protein in rheumatology: Biology and genetics. Nat. Rev. Rheumatol..

[48] Stefaniuk P., Szymczyk A., Podhorecka M. (2020). The Neutrophil to Lymphocyte and Lymphocyte to Monocyte Ratios as New Prognostic Factors in Hematological Malignancies—A Narrative Review. Cancer Manag. Res..

[49] Yamamoto T., Kawada K., Obama K. (2021). Inflammation-Related Biomarkers for the Prediction of Prognosis in Colorectal Cancer Patients. Int. J. Mol. Sci..

[50] Alsaif S.H., Rogers A.C., Pua P., Casey P.T., Aherne G.G., Brannigan A.E., Mulsow J.J., Shields C.J., Cahill R.A. (2021). Preoperative C-reactive protein and other inflammatory *markers* as predictors of postoperative complications in patients with colorectal neoplasia. World J. Surg. Oncol..

[51] Mouliou D.S. (2023). C-Reactive Protein: Pathophysiology, Diagnosis, False Test Results and a Novel Diagnostic Algorithm for Clinicians. Diseases.

[52] Käser S.A., Fankhauser G., Glauser P.M., Toia D., Maurer C.A. (2010). Diagnostic value of inflammation markers in predicting perforation in acute sigmoid diverticulitis. World J. Surg..

[53] Zhu C.l., Wang Y., Liu Q., Li H.r., Yu C.m., Li P., Deng X.-m., Wang J.-f. (2022). Dysregulation of neutrophil death in sepsis. Front. Immunol..

[54] Cao M., Wang G., Xie J. (2023). Immune dysregulation in sepsis: Experiences, lessons and perspectives. Cell Death Discov..

[55] Branescu C., Serban D., Dascălu A.M., Oprescu S.M., Savlovschi C. (2013). Interleukin 6 and lipopolysaccharide binding protein—Markers of inflammation in acute appendicitis. Chirurgia.

[56] Ji Y., Fish P.M., Strawn T.L., Lohman A.W., Wu J., Szalai A.J., Fay W.P. (2014). C-Reactive Protein Induces Expression of Tissue Factor and Plasminogen Activator Inhibitor-1 and Promotes Fibrin Accumulation in Vein Grafts. J. Thromb. Haemost. JTH.

[57] Buonacera A., Stancanelli B., Colaci M., Malatino L. (2022). Neutrophil to Lymphocyte Ratio: An Emerging Marker of the Relationships between the Immune System and Diseases. Int. J. Mol. Sci..

[58] Wu L., Saxena S., Singh R.K. (2020). Neutrophils in the Tumor Microenvironment.

[59] Koc D.C., Mănescu I.B., Mănescu M., Dobreanu M. (2024). A Review of the Prognostic Significance of Neutrophil-to-Lymphocyte Ratio in Nonhematologic Malignancies. Diagnostics.

[60] Duque-Santana V., López-Campos F., Martin-Martin M., Valero M., Zafra-Martín J., Couñago F., Sancho S. (2023). Neutrophil-to-Lymphocyte Ratio and Platelet-to-Lymphocyte Ratio as Prognostic Factors in Locally Advanced Rectal Cancer. Oncology.

[61] Hu Z., Tan S., Chen S., Qin S., Chen H., Qin S., Huang Z., Zhou F., Qin X. (2020). Diagnostic value of hematological parameters platelet to lymphocyte ratio and hemoglobin to platelet ratio in patients with colon cancer. Clin. Chim. Acta.

[62] Song G., Ren J., Stojadinovic A., Chen W., Sahab Z., Fu S.W., Man Y.G. (2012). Conjunction of tumor cells with lymphocytes: Implications for tumor invasion and metastasis. Cancer Epidemiol..

[63] Rees P.A., Clouston H.W., Duff S., Kirwan C.C. (2018). Colorectal cancer and thrombosis. Int. J. Colorectal Dis..

[64] Partl R., Paal K., Stranz B., Hassler E., Magyar M., Brunner T.B., Langsenlehner T. (2023). The Pre-Treatment Platelet-to-Lymphocyte Ratio as a Prognostic Factor for Loco-Regional Control in Locally Advanced Rectal Cancer. Diagnostics.

[65] Rahimi M., Ansari M., Abdollahi A., Gholinezhadan A., Taherynezhad M., Zarif-Sadeghian M., Tabatabaei S.M., Shahabi F. (2025). A comprehensive feature importance analysis of surgical site infection following colorectal cancer surgery. Sci. Rep..

[66] Verras G.I., Mulita F. (2024). Butyrylcholinesterase levels correlate with surgical site infection risk and severity after colorectal surgery: A prospective single-center study. Front. Surg..

[67] Mulita F., Akinosoglou K., Velissaris D., Panos G., Maroulis I. (2022). Correlation between surgical site infection and TNM classification of malignant tumors after colorectal surgery: A single-center prospective study. Eur. J. Surg. Oncol..

[68] Koperna T., Semmler D., Marian F. (2001). Risk Stratification in Emergency Surgical Patients: Is the APACHE II Score a Reliable Marker of Physiological Impairment?. Arch. Surg..

[69] Mulita F., Verras G.I., Bouchagier K., Dafnomili V.D., Skroubis G., Perdikaris I., Perdikaris P., Velissaris D., Perdikaris P., Velissaris D. (2023). The simultaneous use of APACHE II and NEWS II clinical scores in postoperative monitoring of the colon and rectum surgical patient. Eur. J. Surg. Oncol..

[70] Chen W.S., Lin J., Zhang W.T., Chen W.J., Gabriel E.M., Kuo P.C., Caycedo-Marulanda A., Cai Y.-Q., Chen X.D., Wu W.Y. (2023). Effect of low-level creatinine clearance on short-term postoperative complications in patients with colorectal cancer. J. Gastrointest. Oncol..

[71] Mannion J.D., Rather A., Fisher A., Gardner K., Ghanem N., Dirocco S., Siegelman G. (2024). Systemic inflammation and acute kidney injury after colorectal surgery. BMC Nephrol..

[72] Yang T., Richards E.M., Pepine C.J., Raizada M.K. (2018). The gut microbiota and the brain-gut-kidney axis in hypertension and chronic kidney disease. Nat. Rev. Nephrol..

[73] Pacilli M., Fersini A., Pavone G., Cianci P., Ambrosi A., Tartaglia N. (2022). Emergency Surgery for Colon Diseases in Elderly Patients—Analysis of Complications, and Postoperative Course. Medicina.

[74] Garcia J.R., Allende F., Kogan M., Chahla J. (2024). When Things Go Wrong: A Guide to the Medical, Ethical, and Legal Dimensions of Surgical Complications. JAAOS Glob. Res. Rev..

